# More than additive effects on liver triglyceride accumulation by combinations of steatotic and non-steatotic pesticides in HepaRG cells

**DOI:** 10.1007/s00204-021-02997-2

**Published:** 2021-02-11

**Authors:** Alexandra Lasch, Philip Marx-Stoelting, Albert Braeuning, Dajana Lichtenstein

**Affiliations:** 1 Department of Food Safety, German Federal Institute for Risk Assessment, Max-Dohrn-Straße 8-10, 10589 Berlin, Germany; 2 Department of Pesticides Safety, German Federal Institute for Risk Assessment, Max-Dohrn-Straße 8-10, 10589 Berlin, Germany

**Keywords:** Mixtures, Adverse outcome pathway, Liver steatosis, Nuclear receptor activation, Pesticides

## Abstract

The liver is constantly exposed to mixtures of hepatotoxic compounds, such as food contaminants and pesticides. Dose addition is regularly assumed for mixtures in risk assessment, which however might not be sufficiently protective in case of synergistic effects. Especially the prediction of combination effects of substances which do not share a common adverse outcome (AO) might be problematic. In this study, the focus was on the endpoint liver triglyceride accumulation in vitro, an indicator of hepatic fatty acid changes. The hepatotoxic compounds difenoconazole, propiconazole and tebuconazole were chosen which cause hepatic fatty acid changes in vivo, whereas fludioxonil was chosen as a hepatotoxic substance not causing fatty acid changes. Triglyceride accumulation was analyzed for combinations of steatotic and non-steatotic pesticides in human HepaRG hepatocarcinoma cells. Investigations revealed a potentiation of triglyceride accumulation by mixtures of the steatotic compounds with the non-steatotic fludioxonil, as compared to the single compounds. Mathematical modeling of combination effects indicated more than additive effects for the tested combinations if the method by Chou was applied, and a decrease in EC_50_ values of the steatotic compounds when applied in mixtures. Use of an adverse outcome pathway (AOP)-driven testing strategy for liver steatosis showed interactions of the test compounds with the nuclear receptors AHR, CAR and PXR, as well as a downregulation of *ACOX2*. An ACOX2-dependent mechanism underlying the observed mixture effect could not be verified using a siRNA approach. By contrast, a toxicokinetic interaction was identified including an inhibition of the metabolic enzyme CYP3A4 by fludioxonil and a decreased metabolic conversion of the CYP3A4 substrate difenoconazole when used in mixture experiments. In conclusion, an interaction by a steatotic and a non-steatotic compound at the toxicokinetic level on the endpoint triglyceride accumulation in vitro was described.

**Supplementary Information:**

The online version contains supplementary material available at 10.1007/s00204-021-02997-2.

## Introduction

The constant exposure of consumers to mixtures of chemicals is of concern for risk assessment as they could, in principle, cause toxic effects in humans different from the effects of individual compounds. Risk assessment is mostly done for single compounds and thus might not accurately reflect the actual risks resulting from multiple exposures. Biological effects that may occur with a chemical mixture can be classified into additive, antagonistic or synergistic effects (ECHA 2017; More et al. 2019; OECD 2018; Rotter et al. 2018), whereby especially synergistic effects are of concern to the consumer as the general assumption for risk assessment of mixtures is dose addition. Given the almost infinite number of possible combinations, the appropriate evaluation of the interaction of substances in vitro is an important topic in food toxicology that warrants further research. Essential for the assessment of mixtures is information about the mode of action of the individual substances, as this information may help predicting possible overadditive effects.

Liver steatosis is a widespread disease and has gained more and more attention over the last years (Perumpail et al. 2017). It is characterized by an increased intracellular triglyceride content in hepatocytes. Several factors may participate in the development of steatosis, such as toxicants and drugs (Al-Eryani et al. 2015; Fromenty and Pessayre 1997; Joshi-Barve et al. 2015). Vinken (2015) and Mellor et al. (2016) depicted the mechanism of this toxicological process in an adverse outcome pathway (AOP). AOPs link a molecular initiating event (MIE) with key events (KE) which then lead to an adverse outcome (AO) (Ankley et al. 2010; Villeneuve et al. 2014). Nuclear receptor (NR) activation is named as the molecular initiating event (MIE) in the steatosis AOP. NRs involved in the progression of steatosis are the aryl hydrocarbon receptor (AHR), constitutive androstane receptor (CAR), estrogen receptor (ER), farnesoid-X-recptor (FXR), glucocorticoid receptor (GR), liver-X-receptor (LXR), peroxisome proliferator-activated receptor (PPAR) alpha, pregnane-X-receptor (PXR), retinoic acid receptor (RAR) and retinoid-X-receptor (RXR) (Mellor et al. 2016; Vinken 2015). The interaction of chemical agonists with those receptors leads to several intermediate effects like altered gene expression (*MLXIPL*, *SREBF1*, *SCD*, *FASN*, *CD36*), increase of fatty acid influx, de novo synthesis of fatty acids, inhibition of respiration and mitochondrial/microsomal β-oxidation and finally to liver triglyceride accumulation (Mellor et al. 2016).

Aim of this study was to evaluate if combination effects on liver triglyceride accumulation may occur when substances which do not share the same AO are combined. Therefore, analysis of triglyceride accumulation was performed using combinations of steatotic and non-steatotic substances in the human hepatocarcinoma cell line HepaRG. Previous studies already revealed that HepaRG cells are an appropriate model to study steatosis in vitro (Antherieu et al. 2010; Knebel et al. 2019a; Lasch et al. 2020a; Luckert et al. 2018; Tanner et al. 2018; Tolosa et al. 2016). The following test substances were chosen: fludioxonil (FDO) as a non-steatotic substance, as well as difenoconazole (DIF), propiconazole (PPC) and tebuconazole (TBC) as steatotic substances. Within the cumulative assessment groups (CAG) for pesticides established by the European Food Safety Authority EFSA, FDO is listed as toxic to the liver but not reported to cause hepatic fatty changes. In contrast, the three triazoles DIF, PPC and TBC are described as toxic to the liver and to cause fatty changes in the liver (Nielsen et al. 2012).

## Materials and methods

### Chemicals

Difenoconazole (DIF) (1-[[2-[2-chloro-4-(4-chlorophenoxy)phenyl]-4-methyl-1,3-dioxolan-2-yl]methyl]-1,2,4-triazole) (CAS 119446–68-3; batch: BCBS9001V; purity: ≥ 99%) and fludioxonil (FDO) (4-(2,2-difluoro-1,3-benzodioxol-4-yl)-1*H*-pyrrole-3-carbonitrile) (CAS 131341–86-1; batch: BCBV2209; purity: ≥ 99%) were obtained from Sigma-Aldrich (Taufkirchen, Germany). Propiconazole (PPC) (1-[[2-(2,4-dichlorophenyl)-4-propyl-1,3-dioxolan-2-yl]methyl]-1,2,4-triazole) (CAS 60207–90-1; batch:G144536; purity: 99.0%) and tebuconazole (TBC) (1-(4-chlorophenyl)-4,4-dimethyl-3-(1,2,4-triazol-1-ylmethyl)pentan-3-ol) (CAS 107534–96-3; batch: G142375; purity: 99.6%) were obtained from LGC Standards (Wesel, Germany).

### Cultivation of HepaRG and HepG2 cells

HepaRG cells (Biopredic International, Sant Grégoire, France) were cultivated 4 weeks before they were used for experiments (Gripon et al. 2002): cultivation was divided into two weeks of proliferation and two weeks of differentiation. Cells were seeded in 96-well plates at a density of 9000 cells/well and in 12-well plates at a density of 100,000 cells/well. Proliferation was achieved in William’s medium E with 2 mM glutamine (PAN-Biotech, Aidenbach, Germany) 10% fetal bovine serum (FBS; FBS Good Forte EU approved; PAN-Biotech, Aidenbach, Germany), 100 U/mL penicillin and 100 µg/mL streptomycin (Capricorn Scientific, Ebsdorfergrund, Germany), 0.05% human insulin (PAN-Biotech, Aidenbach, Germany) and 50 µM hydrocortisone-hemisuccinate (Sigma-Aldrich, Taufkirchen, Germany). Differentiation medium consisted of proliferation medium with additional 1.7% dimethylsulfoxide (DMSO). 48 h before experiments, medium of differentiated HepaRG cells was changed to induction medium, containing only 2% FBS and 0.5%DMSO.

HepG2 (ECACC, Salisbury, UK), a human hepatocellular carcinoma cell line, was cultivated in Dulbecco’s modified Eagle’s medium (DMEM; Pan-Biotech, Aidenbach, Germany). DMEM was supplemented with 10% FBS (FBS Capricorn Scientific, Ebsdorfergrund, Germany) and 100 U/mL penicillin and 100 µg/mL streptomycin (Capricorn Scientific, Ebsdorfergrund, Germany). By the time cells reached a confluence of 80%, they were passaged or seeded at a density 22,000 cells/well in 96-well plates. During treatment, the medium contained 0.5% DMSO. Both cell lines were cultivated at 37 °C and 5% CO_2_ in a humidified atmosphere.

### Cell viability test

The WST-1 assay (Hoffmann-La Roche, Basel, Switzerland) was used for analysis of cytotoxic effect of the test compounds. Cytotoxic effects of test compounds in HepaRG cells were estimated after 72 h of incubation and in HepG2 cells after 24 h of incubation in 96-well format. 10 µL WST-1 reagent was added to each well one hour before the end of incubation. Absorbance of WST-1 was measured at 450 nm together with a reference wavelength of 620 nm one hour after addition to the cells.

### Triglyceride measurement

AdipoRed reagent (Lonza, Basel, Switzerland) was used to measure the intracellular amount of triglycerides in HepaRG cells after 72 h of incubation. The assay was performed in 96-well plates with 6 technical replicates for each test concentration. Prior to fluorescence measurement, cells were washed with 200 µL phosphate-buffered saline (PBS) and after this, 200 µL PBS containing 5 µg/mL Hoechst 33,342 (Thermo Fisher Scientific, Waltham, Massachusetts, USA) was added to each well. At last, 5 µL AdipoRed reagent was added. 96-well plates were incubated for additional 10 min at 37 °C. Measurement of fluorescence was done with an excitation wavelength of 485 nm and emission at 572 nm for AdipoRed, and with excitation at 350 nm and emission at 461 nm for Hoechst 33,342. To compensate variations in cell numbers, AdipoRed signals were normalized to Hoechst 33,342 signals.

### EROD activity

CYP1A1/CYP1A2 activity was investigated by ethoxyresorufin-*O*-deethylase (EROD) reaction. First, HepaRG cells were incubated for 24 h with the chosen test substances. 5 µM 3-methylcholanthrene was used as positive control. After incubation, cells were washed with PBS and then incubated for 30 min with induction medium with 2 µM ethoxy-resorufin. The concentration of resorufin in the supernatant was determined by fluorescence measurement with excitation at 535 nm and emission at 590 nm. Resorufin concentration was normalized to the protein content; therefore, protein content was measured using the Bicinchoninic Acid Kit for protein determination (Sigma-Aldrich, Munich, Germany).

### ACOX2 knockdown

*ACOX2* knockdown was performed via siRNA. 48 h before transfection, cells were adapted to induction medium. Cells were then transfected twice in an interval of 24 h. A medium exchange with induction medium was performed before every transfection. Transfection procedure was done according to the manual of the transfection reagent lipofectamine RNAiMAX (catalog number: 13778150; Thermo Fisher Scientific, Waltham, MA, USA). The experimental setup included a medium control without any transfection, a control siRNA transfection (Silencer Select Negative Control 1 siRNA, catalog number: 4390844; Thermo Fisher Scientific, Waltham, MA, USA) and a transfection using a mixture of 4 different siRNAs against *ACOX2* (FlexiTube GeneSolution GS8309 for *ACOX2*: SI0432249, SI04304258, SI00291004 and SI00290997; Qiagen, Hilden, Germany). *ACOX2* knockdown was verified at the gene expression and protein levels. The established knockdown was used in combination with the AdipoRed assay. Therefore, the knockdown efficiency was checked 24 h after the second transfection, which was chosen as the starting time for AdipoRed assay incubation and 96 h after the second transfection, as the incubation time of the AdipoRed assay was set to 72 h.

### Carnitin shuttle transporter assay

The carnitine shuttle transporter assay was conducted by SOLVO (SOLVO biotechnology; a Charles River company, Budapest, Hungary) under subcontract according to a standard protocol. In brief, solubility of all substances was checked in assay buffer at 37 °C. Substances were applied up to max soluble concentration in two different assays: in an uptake transporter assay, the accumulation of the probe substrate in the presence of test substance (inhibition) and/or the accumulation of the test substance (substrate) into cells was measured. The test article, reference inhibitor and solvent control were tested in transporter-expressing cells in triplicates. Transporter-expressing cells were cultured according to the general SOLVO protocol (PR-CC-UPT-General Protocol for Culturing Uptake Cell Lines).

### Dual-luciferase reporter assay

The assays were performed as previously described by Luckert et al. (2018). Plasmids, plasmid amount and positive controls were also used as described in the aforementioned paper. In brief, HepG2 cells were used to measure activation of NRs (AHR, CAR, FXR, GR, LXR, PPARα, PPARγ, PPARδ, PXR, RAR and RXR) via dual-luciferase reporter assays. Cells were transfected 24 h after seeding with the plasmids for 4–6 h using TransIT-LT1 (Mirus Bio LCC, Madison, WI, USA). Only the AHR reporter plasmid was transfected during cell seeding as this resulted in better luciferase signals. After 4–6 h of transient transfection (or after 24 h for AHR), the cells were incubated with the chosen test compounds in culture medium containing 0.5% DMSO. Cells were lysed with 50 µL lysis buffer (100 mM potassium phosphate with 0.2% (v/v) Triton X-100, pH 7.8). 5 µL of the cell lysate was investigated. Luminescence was measured for firefly and *Renilla* luciferase activities in a dual luciferase assay with an Infinite M200 Pro plate reader (Tecan group, Männedorf, Switzerland), as previously described by Hampf and Gossen (2006). The firefly signal indicates an interaction with the NR, whereas the *Renilla* signal is used as an internal control for normalization as the plasmid is constitutively expressed by the cells.

### Gene expression analysis

Incubation for gene expression analysis was performed in 12-well plate. After 24 h of incubation with the test substances, cells were washed twice with PBS and RNA was isolated using the RNeasy Mini Kit (Qiagen, Hilden, Germany) according to manufacturer’s instructions. RNA concentration and purity were determined by Infinite M200 Pro plate reader (Tecan group, Männedorf, Switzerland) at wavelengths of 260 nm and 280 nm. According to the manufacturer’s instructions, 1000 ng RNA was used for cDNA synthesis with the High Capacity cDNA Reverse Transcription Kit (Applied Biosystems, Darmstadt, Germany). Quantitative real-time RT-PCR was performed using Maxima SYBR Green/ROX qPCR Master Mix (Thermo Fisher Scientific, Waltham, MA, USA), 20 ng cDNA, primers (5 µM; see Table 1) and an AriaDx Realt-Time PCR Instrument (Agilent Technologies, Santa Clara, CA, USA). Thermal cycling conditions were used as previously described by Luckert et al. (2013). Results were evaluated using the ∆∆Ct method (Livak and Schmittgen 2001). *ACTB* and *GAPDH* were used as reference genes as they were found to be stably expressed throughout treatments (supplementary data, Fig. S1). Their geometric mean was used for normalization.

**Table 1:** Sequences of real-time RT-PCR primers

Gene	Accession number	Forward primer (5′–3′)	Reverse primer (5′–3′)
*ACTB*	ENSG00000075624	CGTCCACCGCAAATGCTT	GTTTTCTGCGCAAGTTAGGTTTTGT
*GAPDH*	ENSG00000111640	ATTTGGCTACAGCAACAGGG	CAACTGTGAGGAGGGGAGA
*ACOX1*	ENSG00000161533	CTGAAGGCTTTCACCTCCTG	GGCAGGTCGTTCAAATAGGA
*ACOX2*	ENSG00000168306	GCTTACAGAGCCCTTTCTGGAG	AAGTCTCCAGGCCACCATTTG
*AHR*	ENSG00000106546	CCAGACCAGATTCCTCCAGA	TTCATTGCCAGAAAACCAGA
*CD36*	ENSG00000135218	TGATGAACAGCAGCAACATTC	CAGCGTCCTGGGTTACATTT
*CYP1A1*	ENSG00000140465	ACCCTGAAGGTGACAGTTCC	TCTTGGAGGTGGCTGAGGTA
*CYP1A2*	ENSG00000140505	CTTCGCTACCTGCCTAACCC	CCCGGACACTGTTCTTGTCA
*CYP2B6*	ENSG00000197408	TTCGGCGATTCTCTGTGACC	ATGAGGGCCCCCTTGGAT
*CYP3A4*	ENSG00000160868	TCAGCCTGGTGCTCCTCTATCTAT	AAGCCCTTATGGTAGGACAAAATATTT
*FASN*	ENSG00000169710	ACAGCGGGGAATGGGTACT	GACTGGTACAACGAGCGGAT
*MLXIPL*	ENSG00000009950	GCCTGAACAACGCCATCT	GGTCACGAAGCCACACAC
*NR1I2 (PXR)*	ENSG00000144852	GGCATGAAGAAGGAGATGAT	TGGGAGAAGGTAGTGTCAAA
*NR1I3 (CAR)*	ENSG00000143257	ATGCTGGCATGAGGAAAGAC	GTTGCACAGGTGTTTGCTGT
*SCD*	ENSG00000099194	ACCGCTGGCACATCAACTTC	CCTTGGAGACTTTCTTCCGGTC
*SREBF1*	ENSG00000072310	CGGAACCATCTTGGCAACAGT	CGCTTCTCAATGGCGTTGT

### Western blotting

For protein extraction, cells were washed with PBS and harvested with RIPA buffer (radioimmunoprecipitation assay buffer) with protease and phosphatase inhibitors. Protein extraction was performed on ice via sonication (two times for 10 s with 10% cycles; 25% power; Sonopuls UW 2200, Bandelin Electronic GmbH & Co. KG, Berlin, Germany). After sonication, samples were centrifuged at 4 °C, 20,817×*g* for 30 min. The supernatant, containing the proteins, was used for protein analysis. Protein content was measured using the Bicinchoninic Acid Kit for protein determination (Sigma-Aldrich, Munich, Germany). 20 µg of protein was heated to 95 °C for 5 min and separated on 10% sodium dodecylsulfate polyacrylamide gels (BioRad Mini PROTEAN Tetra System, Bio-Rad Laboratories, Inc., Hercules, CA, USA). A semi-dry method (TE 77 PWR Semi-Dry Transfer Unit, 21 × 26 cm; Amersham Biosciences, GE Healthcare GmbH, Solingen, Germany) was used to blot the separated proteins to nitrocellulose membranes. Membranes were blocked for 2 h at room temperature with 5% milk powder in Tris-buffered saline with 1% (v/v) Tween 20 (TBST buffer). Membranes were incubated over night at 4 °C with the primary antibody against ACOX2 (1:100, in TBST with 5% milk powder, ACOX2 (A-7): sc-514320; Santa Cruz Biotechnology, Dallas, TX, USA). The secondary antibody (Anti-mouse-IgG-HRP-conjugated: A-014HRP; Seramun Diagnostica, Heidensee, Germany) was diluted 1:7500 in TBST with 5% milk powder and incubated with the membranes for 1 h at room temperature. As housekeeping control, pan-actin antibody (Lab Vision Actin, pan Ab-5, Mouse Monoclonal Antibody: MS-1295-P; ThermoFisher Scientific, Waltham, MA, USA) was used and diluted 1:5000 in TBST with 5% milk powder. The housekeeping antibody was incubated for 1 h at room temperature with the membranes and the same secondary antibody as described before was used. Detection was done using the Super Signal West Femto Maximum Sensitivity Substrat Kit (ThermoFisher Scientific, Waltham, MA, USA) and a VersaDocTM Mp 4000 system equipped with the Quantity One Software (Vers. 4.6.1; Bio-Rad Laboratories, Inc., Hercules, CA, USA).

### Pesticide quantification in cell culture supernatant

For the quantification of the test substances in cell culture supernatant, the substances were incubated with and without HepaRG cells for 8 h at 37 °C and 5% CO_2_ in a humidified atmosphere. The incubation was performed in 12-well plates. After 8 h, the medium was harvested and cells were washed with ultrapure water which was added to the harvested medium. Samples were diluted with ultrapure water to 21 mL sample volume. Measurement of DIF and FDO was performed by a certified commercial laboratory (SGS, Institute Fresenius, Berlin, Germany) according to the method laid down in DIN EN 15,662 (ASU L00.00–115). The accredited method comprises a QuEChERS solid-phase extraction method and LC–MS/MS quantification.

### Measurement of CYP3A4 enzyme activity

CYP3A4 enzyme activity was measured using the luminogenic CYP3A4 substrate Luciferin-IPA from Promega (Madison, Wisconsin, USA) (catalog number: V840A). The substrate was incubated with insect control supersomes (Corning, New York, USA) or human CYP3A4 supersomes from Corning (catalog number: 456202) (Corning, New York, USA). The latter convert the substrate to d-luciferin. Detection was performed with the luciferin detection reaction (catalog number: V859A) dissolved in reconstitution buffer with esterase (catalog number: V144A) from Promega (Madison, Wisconsin, USA). Experiments were performed in white 96-well plates in triplicates. For one well 7.35 µL water, 5 µL sodium phosphate buffer (1 M, pH 7.4), 0.05 µL Luciferin-IPA (3 mM), and 0.1 µL CYP3A4 supersomes were mixed. Experiments were conducted by adding 12.5 µL of test compound (4 × concentrated to compensate for the dilution effect in the assay), or a CYP3A4 inhibition control (ketoconazole, final concentration 1 µM) or water as negative control to each well. After this, 12.5 µL of the mix with control supersomes as negative control or CYP3A4 supersomes was added, mixed on a plate shaker and pre-incubated for 10 min at 37 °C. The reaction was started by adding 25 µL of cofactor solution, containing 33 mM potassium chloride, 8 mM magnesium chloride, 1 mM NADP and 5 mM glucose-6-phosphate. Plate was again shaken and incubated for 30 min at 37 °C. Finally, 50 µL of luciferin detection reagent was added to each well and the plate was incubated for 20 min at room temperature. After this, luminescence was measured with 1 s integration time.

### Evaluation of combination effects

The evaluation of combination effects was performed with three mathematical models: the theoretical additivity method (TA), the concentration addition concept (CA) and the independent action concept (IA). With the help of these models, a mixture effect was calculated based on the single compound effects. The calculated mixture effect refers to a dose addition assumption. Detailed description on the calculations with the different models can be found in the publication by Lasch et al. (2020a). To evaluate the combination effects, the combination index (CI) was calculated as described by Foucquier and Guedj (2015):

$${\text{CI}} = \frac{{\text{calculated mixture effect}}}{{\text{measured mixture effect}}}$$

A CI < 0.9 indicates synergism, a CI between 0.9 and 1.1 indicates an additive effect and a CI > 1.1 indicates antagonism (Chou 2006). Another threshold considered to differ between additivity, antagonism and synergism is the model deviation ratio (MDR). The MDR has the same mathematical definition as the CI, but has different thresholds. An MDR < 0.5 indicates synergism, an MDR between 0.5 and 2 indicates an additive effect and an MDR > 2 indicates antagonism (Belden et al. 2007; Cedergreen 2014).

### Statistics

Statistical analysis was performed using SigmaPlot software (Version 14.0). Parametric statistical tests were chosen as they are more powerful than non-parametric tests. A one-way ANOVA followed by Dunnett ‘s post hoc test was performed for calculating statistical significance in a concentration series against the medium control with *p ≤ 0.05, **p ≤ 0.01 and ***p ≤ 0.001. Scenarios with calculations of statistical differences of mixtures and single compounds were performed using one-way ANOVA followed by the Holm–Sidak post hoc test (all pairwise testing) with *p ≤ 0.05, **p ≤ 0.01 and ***p ≤ 0.001 for concentration series of single compounds; ^#^*p* ≤ 0.05, ^##^*p* ≤ 0.01 and ^###^*p* ≤ 0.001 for concentration series of mixtures and + *p* ≤ 0.05, ++ *p* ≤ 0.01 and +++*p* ≤ 0.001 for comparison of mixtures and single compounds at same concentration levels. EC_50_ calculation and curve fitting was also performed using SigmaPlot software (Version 14.0).

## Results

### More than additive effects of triglyceride accumulation by mixtures

**Fig. 1:**
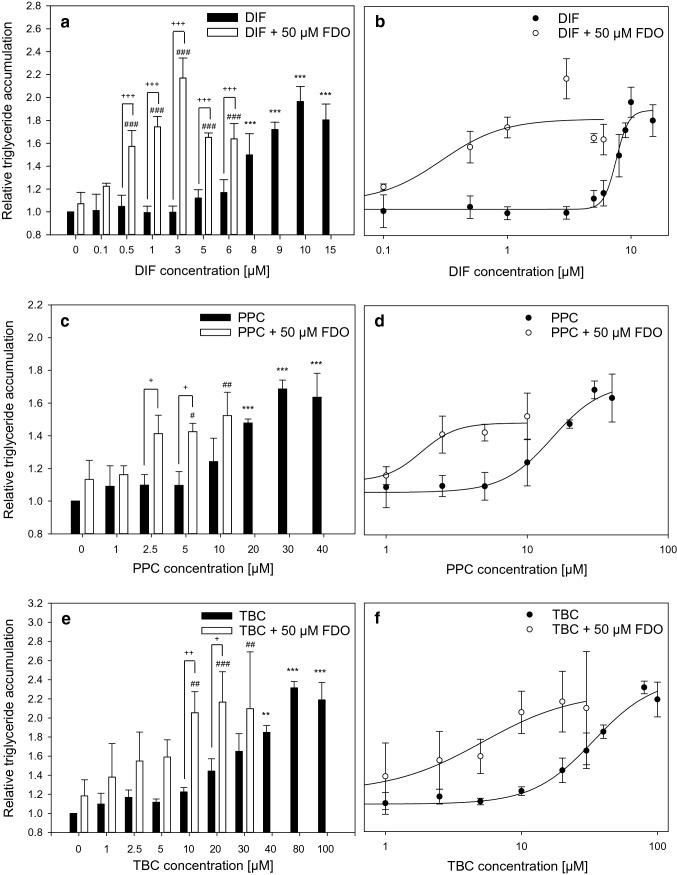
Triglyceride accumulation in HepaRG cells induced by DIF, PPC, TBC alone and their mixtures with 50 µM FDO (**a**, **c**, **e**). Intracellular triglycerides were measured with the AdipoRed assay after 72 h of incubation. **b**, **d** and **f** show the data points of DIF, PPC, TBC alone and their mixtures with 50 µM FDO, respectively, and the fitted curves for EC_50_ calculation. Cyproconazole (200 µM) was used as positive control (data not shown). Data represent mean ± SD (N ≥ 3 independent experiments). Statistic was done by one-way ANOVA with Holm–Sidak post hoc test (all pairwise): *p ≤ 0.05, **p ≤ 0.01 and ***p ≤ 0.001 indicate significance for single compounds (black bars) against the medium control containing 0.5% DMSO, #*p* ≤ 0.05, ##*p* ≤ 0.01 and ###*p* ≤ 0.001 indicate significance for combinations (white bars) against 0 µM DIF/PPC/TBC + 50 µM FDO, while + *p* ≤ 0.05, ++ *p* ≤ 0.01 and +++ *p* ≤ 0.001 indicate significance for single compounds (black bars) against combinations (white bars) at the same concentrations of DIF/PPC/TBC

The aim of this study was to investigate binary mixtures of steatotic and non-steatotic compounds and thus the influence of a non-steatotic compound on steatosis caused by the steatotic substance. As the tested combinations do not share the same outcome with respect liver triglyceride accumulation, we decided to analyze mixtures with increasing concentrations of the steatotic compounds and constant concentrations of the non-steatotic compound. Therefore, FDO (i.e., the non-steatotic compound) was combined at a constant concentration of 50 µM with the steatotic substances DIF, PPC and TBC, which were applied at increasing concentrations. Initial analysis of cytotoxicity was performed for all test compounds and their mixtures to ensure the use of non-toxic concentrations in all experiments (data not shown). Triglyceride content of HepaRG cells was measured after 72 h of incubation with the chosen test compounds. Figures 1a, c, e show the induction of triglyceride accumulation of the single compounds DIF, PPC and TBC and of their mixtures with FDO. All three single steatotic substances increased the triglyceride content in HepaRG cells in a concentration-dependent manner. FDO alone did not cause a significant increase in triglyceride accumulation. The three combinations of FDO with one of the triazoles resulted in a potentiation of the steatotic effect of the single compounds. When combined with FDO, the triglyceride content of HepaRG cells increased already at low triazole concentrations, at which for the single compounds, no effect was observed. Figures 1b, d, f show the curves that have been used for half-maximal effective concentration (EC_50_) calculation and demonstrates the shift of the curves of mixtures to the left, as compared to the curves obtained with the single compounds. The EC_50_ values of the single compounds and of their mixtures with FDO are listed in Table 2. All three mixtures had lower EC_50_ values than the respective steatotic single compounds. In summary, the results demonstrate that FDO, a compound that shows alone no effect on triglyceride accumulation, had a striking influence on this endpoint in mixtures with substances that per se cause triglyceride accumulation.

**Table 2:** EC_50_ values ± SE of the single compounds DIF, PPC and TBC and of their mixtures with 50 µM FDO

	DIF	DIF + 50 µM FDO	PPC	PPC + 50 µM FDO	TBC	TBC + 50 µM FDO
EC_50_ [µM] ± SE	7.6 ± 0.36	0.3 ± 0.33	15.1 ± 3.21	1.8 ± 0.64	33.8 ± 6.13	5.2 ± 2.8

### Evaluation of combination effects reveals more than additive effects

**Fig. 2:**
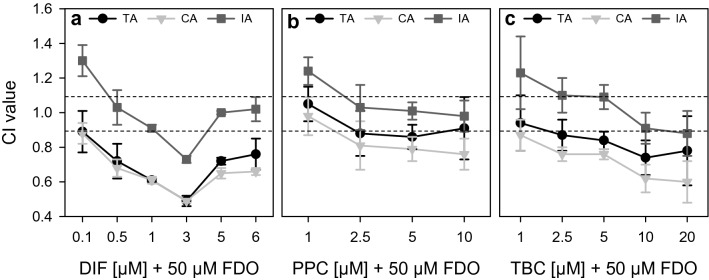
Evaluation of combination effects for triglyceride accumulation caused by DIF (**a**), PPC (**b**) and TBC (**c**) in combination with 50 µM FDO. The effects were analyzed using the TA, IA and CA concepts. CI below 0.9 indicates synergism according to Chou (2006), CI ≈ 1 indicates dose addition, and CI above 1.1 indicates antagonism. The variation of the data is illustrated as for every biological replicate a CI value was calculated to allow the calculation of a SD of the CI values. This shows the variation of the Data represent mean ± SD (*N* = 3 independent experiments).

Figure 1 showed a shift of the concentration–response curve of the mixtures with FDO. To assess the combination effects, mathematical modeling with the TA, IA and CA concepts was performed. To evaluate a combination effect as additive, antagonistic or synergistic, the CI by Chou (2006) was used. All models, displayed in Fig. 2, showed a decrease in the CI value with increasing concentrations of the mixture. Especially for the TA and CA concepts according to Chou (2006), synergistic effects expressed by CI values lower than 0.9 were observed for medium and high concentration levels of the mixtures. The IA concept showed additive and slight overadditive effects. Taken together, the mathematical modeling approaches underlined the occurrence of more than additive effects. Supplementary Fig. S2 shows a comparison of the measured and calculated mixture effects for triglyceride accumulation.

### Activation of steatosis- and metabolism-relevant nuclear receptors

**Fig. 3:**
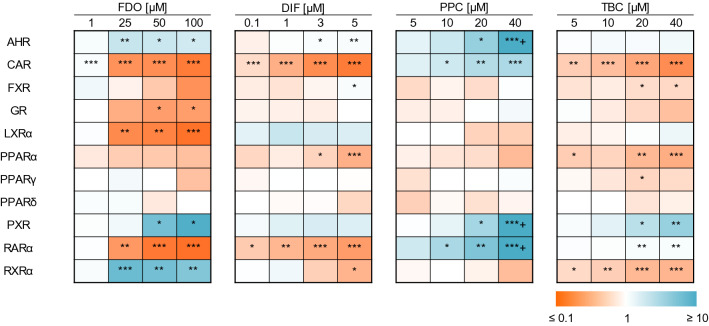
Activation of steatosis-relevant NRs induced by FDO, DIF, PPC and TBC after 24 h incubation in HepG2 cells. Data represent mean fold changes ± SD (*N* ≥ 3 independent experiments). Statistic was done by one-way ANOVA with Dunnett’s test (*p ≤ 0.05, **p ≤ 0.01 and ***p ≤ 0.001) against medium control, + indicates relative luciferase signals lower as 0.1 or higher than 10

As the NR activation is described as the MIE for steatosis, reporter assays for steatosis-relevant NR were performed for the four test compounds FDO, DIF, PPC and TBC (Fig. 3) (detailed values can be found in Supplementary Data Table S1). Those results might give insight into the mode of action of the individual substances and therefore provide first explanations for the potentiation of the steatotic effect. The results revealed that all substances interacted with CAR and PXR: all compounds were agonistic at PXR, even though not statistically significant for DIF. At CAR, only PPC showed an activation, whereas FDO, DIF and TBC lead to an inhibition of the luciferase signals. Another observation was that FDO, DIF and PPC were activating AHR. Those three receptors (AHR, CAR and PXR) are especially participating in the regulation of the biotransformation of xenobiotic compounds and therefore might denote a link to metabolism as underlying cause for the observed mixture effect.

### Regulation of steatosis- and metabolism-related target genes

**Fig. 4:**
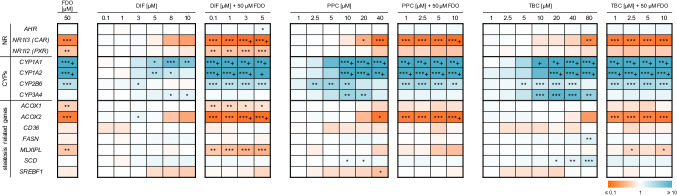
Gene expression analysis after 24 h incubation in HepaRG cells by FDO, DIF, PPC and TBC alone and by mixtures of DIF, PPC and TBC with 50 µM FDO. Data represent means ± SD (*N* = 3 independent experiments). Statistic was done by one-way ANOVA with Dunnett’s test: *p ≤ 0.05, *p ≤ 0.01 and ***p ≤ 0.001 against medium control; + indicates relative expression lower as 0.1 or higher as 10

The NR activation revealed an interaction of the test compounds with AHR, CAR and PXR. Therefore, expression changes of *AHR*, *NR1I3* (*CAR*) and *NR1I2* (*PXR*), their target genes *CYP1A1*, *CYP1A2*, *CYP2B6* and *CYP3A4*, as well as some steatosis-related genes (*ACOX1*, *ACOX2*, *CD36*, *FASN*, *MLXIPL*, *SCD* and *SREPF1*) were investigated to examine if gene expression changes may be responsible for the potentiation of the steatotic effect by FDO. Figure 4 shows the altered gene expression by FDO, DIF, PPC and TBC and by the mixtures of FDO with DIF, PPC and TBC. 50 µM FDO caused an upregulation of *CYP1A1*, *CYP1A2* and *CYP2B6*, whereas *NR1I3* (*CAR*), *NR1I2* (*PXR*), *ACOX1*, *ACOX2* and *MLXIPL* were downregulated. As the effect on *CYP1A1* and *CYP1A2* expression by FDO was very high, we confirmed this result by analyzing CYP1A1/CYP1A2 activity via the EROD reaction. FDO at 50 µM produced a significant increase in EROD activity which is displayed in supplementary data Fig. S3. Most of the genes regulated by FDO were also regulated by DIF, PPC and TBC. Strong combination effects of 50 µM FDO with DIF, PPC or TBC were observed for *NR1I3* (*CAR*), *CYP1A1*, *CYP1A2*, *CYP2B6* and *ACOX2*. These effects are illustrated in supplementary data Fig. S4. *ACOX2* plays an important role in the AOP of steatosis as it is responsible for the degradation of long-branched fatty acids and a downregulation of this gene might therefore be responsible for the accumulation of triglycerides in hepatocytes.

### *ACOX2* downregulation is not responsible for the mixture effect

Gene expression results revealed that 50 µM FDO alone and in the mixtures with DIF, PPC and TBC downregulated *ACOX2* expression, while the steatotic compounds alone did not cause major changes to *ACOX2* expression. ACOX2 is involved in the oxidation of branched-chain fatty acids and a decrease in ACOX2 may lead to the accumulation of branched-chain fatty acids (Baumgart et al. 1996; Ferdinandusse et al. 2018; Vanhove et al. 1993; Vilarinho et al. 2016). Therefore, we hypothesized that FDO contributed to the potentiation of steatosis in the mixtures by diminishing ACOX2 levels, which alone might not have been sufficient to induce steatosis but in combination with the steatosis-promoting molecular effects of the other compounds might have resulted in the observed mixture effects.

**Fig. 5:**
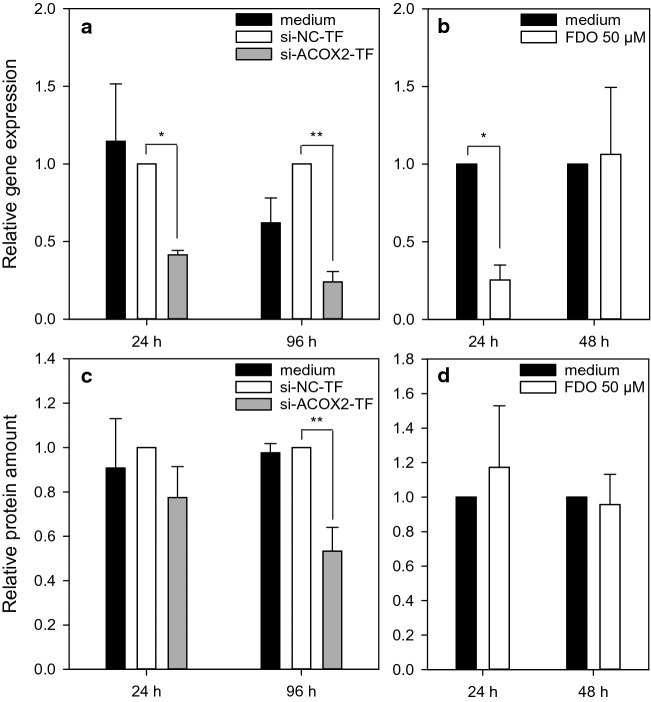
*ACOX2* knockdown via siRNA. HepaRG cells were transfected twice in an interval of 24 h with siRNA for *ACOX2* and with negative control siRNA. Relative gene expression (**a**) and protein amount (**c**) of ACOX2 were analyzed for transfected cells 24 h and 96 h after the second transfection. Relative gene expression (**b**) and protein amount (**d**) of *ACOX2* were also analyzed for cells incubated for 24 h and 48 h with 50 µM FDO. Data represent means ± SD (*N* = 3 independent experiments). Statistic was done by one-way ANOVA with Holm–Sidak post hoc test (all pairwise), *p ≤ 0.05, **p ≤ 0.01 and ***p ≤ 0.001

To investigate whether changes in *ACOX2* expression are responsible for the potentiation of the steatotic effect, a knockdown of *ACOX2* was performed via siRNA instead of incubating the cells with 50 µM FDO. To verify the above hypothesis, such a knockdown should mimic the effects of FDO when combined with DIF, PPC or TBC. Figure 5 shows the results of the establishment of the knockdown. Cells were transfected twice with the siRNA and 24 h and 96 h after the second transfection, the knockdown was verified at the mRNA and protein levels, as the 24 h time point is the starting point of the incubation with the test substances and 96 h is the end of the incubation for the AdipoRed assay. The knockdown of *ACOX2* mRNA already occurred after 24 h and was stable up to 96 h, while the knockdown on protein level was clearly visible after 96 h. For comparison, cells were incubated with 50 µM FDO for 24 h and 48 h. Here, the knockdown was only visible at the gene expression level after 24 h, whereas at the protein level, no knockdown of ACOX2 was detected after incubation with FDO (Fig. 5). Western blot data are included in supplementary data (Fig. S5 and Table S2).

**Fig. 6:**
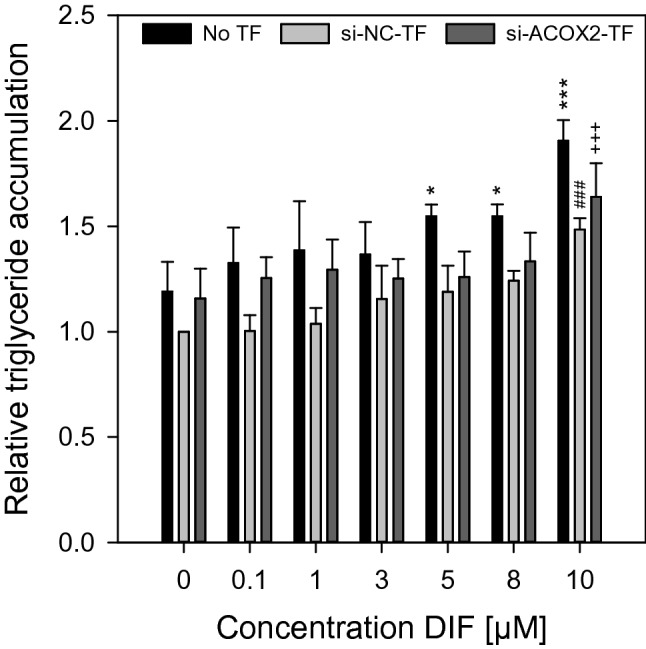
Consequence of *ACOX2* knockdown via siRNA on triglyceride accumulation. HepaRG cells were transfected twice in an interval of 24 h with siRNA for *ACOX2*, or with negative control siRNA. 24 h after the second transfection, 72 h of incubation with DIF were started and the AdipoRed assay was conducted at the end of the incubation period. Relative triglyceride level was measured. Data represent means ± SD (N = 3 independent experiments). Statistic was done by one-way ANOVA with Holm–Sidak post hoc test (all pairwise): (*p ≤ 0.05, **p ≤ 0.01 and ***p ≤ 0.001 for untransfected cells (black bars) against the medium control containing 0.5% DMSO, ^#^*p* ≤ 0.05, ^##^*p* ≤ 0.01 and ^###^*p* ≤ 0.001 for control siRNA-transfected cells (light gray bars) against the medium control containing 0.5% DMSO, or + *p* ≤ 0.05, ++ *p* ≤ 0.01 and +++ *p* ≤ 0.001 for *ACOX2* siRNA-transfected cells (dark gray bars) against the medium control containing 0.5% DMSO

Next, HepaRG cells with a knockdown of *ACOX2* were incubated with the test substance DIF for 72 h, as the mixture of DIF and FDO had revealed the most striking mixture effects for triglyceride accumulation (cp. Fig. 1). Triglyceride accumulation was measured afterwards using the AdipoRed assay. However, triglyceride measurement did not reveal a potentiation of the steatotic effect after knockdown of *ACOX2* (Fig. 6). In general, it was observed that the knockdown procedure itself seemed to slightly affect the assay, as the triglyceride accumulation at same compound concentrations is lower as in non-transfected cells, irrespective of the use of *ACOX2*-specific or negative control siRNA. No significant difference between negative control siRNA-transfected cells and *ACOX2* siRNA-transfected cells was visible. In conclusion, the potentiation effect of the DIF and FDO combination on triglyceride accumulation seemed to be not related to the FDO-mediated decrease in *ACOX2* expression. Another critical key player in fatty acid metabolism is the carnitine shuttle that facilitates uptake of fatty acids in mitochondria for β-oxidation (recently reviewed by Longo et al. (2016)). Since this shuttle is a transporter and some of the substances used in this work are known inhibitors of transporters (e.g. as reviewed by Gubbins and Amsden (2005)), it was reasonable to investigate if the test substances show an inhibitory effect of the carnitine shuttle. Accordingly, the carnitine shuttle was shown to be inhibited by structurally related substances like omeprazol (Pochini et al. 2009). While FDO did not show any inhibition of the carnitine shuttle up to the highest soluble concentration, DIF showed slight inhibitory effect at concentrations above 50 µM (data not shown). No overadditive effect was observed, when both substances were combined in the carnitine shuttle assay.

### Toxicokinetic interactions of FDO and DIF

**Fig. 7:**
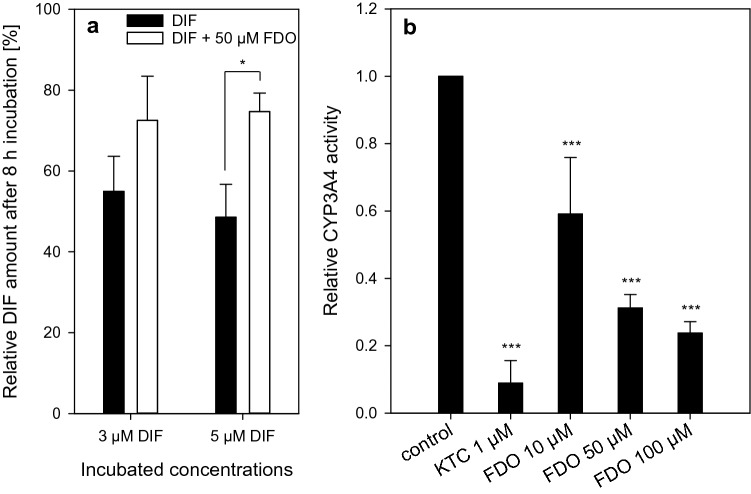
Analysis of toxicokinetic interactions of DIF and FDO. **a** Quantification of DIF in the cell culture supernatant after 8 h of incubation with HepaRG cells. DIF was incubated alone or in the mixture with 50 µM FDO with HepaRG cells. The measured amount of DIF after incubation with HepaRG cells was normalized to the recovery of DIF from control incubations without cells. Data represent means ± SD (*N* = 3 independent experiments). Statistics was done by one-way ANOVA with Holm-Sidak post hoc test (all pairwise) with *p ≤ 0.05. **b** Investigation of CYP3A4 inhibition by FDO. Luciferin-IPA, a specific CYP3A4 substrate, was incubated for 30 min with CYP3A4 supersomes with and without FDO or ketoconazole (KTC). KTC was used as positive control for CYP3A4 inhibition. Treatments with FDO and KTC were normalized to the control without any substance treatment. Statistics was done by one-way ANOVA with Dunnett ‘s test: ***p ≤ 0.001 against control

After having excluded ACOX2 as mediator of the observed mixture effect, the results obtained so far, showing alterations in xenobiotic-metabolizing enzymes, indicated the possibility of a toxicokinetic effect to explain the potentiation of triglyceride accumulation when cells were treated with the mixtures. Therefore, the remaining concentration of DIF was quantified in cell culture supernatant after incubation, either after cell treatment with the single substance or when applied in the mixture with FDO. The combination of DIF and FDO was chosen because it yielded the most prominent mixture effect in the AdipoRed assay. Our hypothesis was that a decrease in DIF metabolism caused by FDO could be a suitable explanation for the observed mixture effects, as a deficiency of DIF metabolism would lead to the presence of more DIF on the cells for a longer period of time in the presence of FDO as compared to single DIF treatment, thereby causing more efficient triglyceride accumulation. The results show that approximately 50% of DIF disappeared from the supernatant in the single-treatment scenario during incubation with the HepaRG cells, whereas in the mixture scenario with FDO only, around 25% of DIF disappeared, suggesting a statistically significant inhibitory effect of FDO on the metabolism of DIF (Fig. 7a). According to literature, DIF is mainly metabolized by CYP3A4 (Wetmore et al. 2014). Therefore, we hypothesized that FDO could be an inhibitor of CYP3A4 enzyme activity. For this reason, a specific luminogenic CYP3A4 substrate was incubated with CYP3A4 supersomes alone and in combination with FDO. The transformation of the substrate was quantified via luminescence measurement. Figure 7b shows a concentration-dependent decrease in CYP3A4 activity caused by FDO. This result underlined our hypothesis that FDO decreases the metabolism of DIF by inhibiting CYP3A4 enzyme activity.

## Discussion

This study was designed to evaluate if combination effects on liver triglyceride accumulation may occur by mixtures of substances which do not share the same AO. For this aim, three triazoles (DIF, PPC and TBC) were selected as steatosis-inducing test compounds. Triazoles are widely used in plant protection products because of their antifungal properties (EFSA 2009). It is known that they can cause adverse effects to mammals, for example, by inhibiting CYP enzymes or via interactions with nuclear receptors followed by alterations in their target gene expression (Marx-Stoelting et al. 2020; Tully et al. 2006). The main target organ for adverse effects caused by most triazoles is the liver (Goetz and Dix 2009; Nesnow et al. 2009). FDO was chosen as non-steatotic compound. FDO, a phenylpyrrole, belongs as well to the group of fungicides and its target organs are liver and kidney (EFSA 2007).

The AdipoRed results in HepaRG cells confirmed the classification of DIF, PPC and TBC into the CAG of hepatocellular fatty changes, and of FDO not being a member of this group of pesticides (Nielsen et al. 2012). Our study revealed a potentiation of triglyceride accumulation in HepaRG cells when the steatotic triazoles DIF, PPC and TBC were combined with the non-steatotic compound FDO. Mathematical modeling of mixture effects classified these results as more than additive. There are different approaches on how to evaluate mixture toxicity and at which CI or MDR an effect should be regarded as synergistic (Belden et al. 2007; Cedergreen 2014; Chou 2006). If a 10% deviation is assumed as proposed by Chou, our results can be described as synergistic. If an MDR of 0.5 is assumed, the observed effects are in most cases not synergistic but still more than additive. This discrepancy illustrates the need for harmonization and specific guidance in this area of increasing regulatory importance as suggested by Lasch et al. (2020b). Furthermore, it has already been shown that mixtures of FDO and triazoles can cause more than additive effects: Wang et al. (2020) showed synergistic toxic effects on mortality of embryonic zebrafish by mixtures of FDO and triadimefon, which also belongs to the group of triazoles. Moreover, it has been reported by Cedergreen (2014) that especially five groups of pesticides were involved in synergistic mixtures, for example, the group of triazoles.

An AOP-oriented in vitro testing strategy was chosen to investigate the molecular basis of the potentiation of liver triglyceride accumulation. Activation of steatosis-relevant NRs was investigated, revealing interactions of the test compounds especially with the NRs AHR, CAR and PXR. For FDO, DIF, PPC and TBC, it has already been shown that these substances can activate PXR (Chaturvedi et al. 2010; de Sousa et al. 2014; Knebel et al. 2019a; Shah et al. 2011). The results by Knebel et al. (2019a) on AHR and CAR activation by PPC and TBC are in line with our present findings. Furthermore, Shah et al. (2011) described an interaction of FDO with AHR and CAR. Gene expression analysis results confirmed the NR interaction of the test compounds, as a deregulation of *CYP1A1*, *CYP1A2*, *CYP2B6* and *CYP3A4*, model NR target genes related to the biotransformation of xenobiotic compounds, was shown. FDO upregulated the expression of several CYPs and downregulated the steatosis-related gene *ACOX2*. These gene expression changes were also visible for the mixtures with FDO, thus leading to hypotheses to explain the potentiation of the steatotic effect of triazoles by FDO.

The first hypothesis relates to the expression of *ACOX2*. ACOX2 is involved in the oxidation of branched-chain fatty acids and a decrease in *ACOX2* expression may lead to the accumulation of branched-chain fatty acids, such as phytanic and pristanic acid (Baumgart et al. 1996; Ferdinandusse et al. 2018; Vanhove et al. 1993; Vilarinho et al. 2016). The steatotic compounds DIF, PPC and TBC also tended to decrease *ACOX2* gene expression, but only at high compound concentrations, whereas FDO appeared to be a potent inhibitor of *ACOX2* expression. For this reason, *ACOX2* was decreased at low concentrations of DIF, PPC and TBC in the mixture experiments, constituting a possibly pro-steatotic molecular event occurring in addition to the effects caused by the triazoles. Thus, even if diminished ACOX2 levels alone, as caused by FDO treatment, are assumed to be not sufficient for steatosis induction, the combination of the molecular effects of triazoles with *ACOX2* inhibition by FDO might cause a more than additive effect, driven by toxicodynamic synergies of the different compounds. However, this hypothesis could not be confirmed based on the data obtained with the *ACOX2* siRNA approach.

The second hypothesis relates to toxicokinetic effects. Induction or inhibition of xenobiotic-metabolizing enzymes by one substance in the mixture can either increase or decrease the metabolism of other compounds (Hernández et al. 2017). Some triazoles are used as first-line drugs for the treatment of systemic mycoses and are therefore often prescribed in combination with other drugs. Because of their inhibitory effects on, for example, CYP3A4, drug–drug interactions are likely and might result in adverse drug reactions (Cai et al. 2020). As mentioned above, triazoles have been reported to belong to one of five groups of pesticides which are overrepresented in synergistic mixtures. Their synergistic potential is thought to be attributed to toxicokinetic effects, such as inhibition of metabolism (Cedergreen 2014). *ACOX2* downregulation was shown to be not responsible for the potentiation effect on triglyceride accumulation, but our results point towards the second hypothesis dealing with a toxicokinetic effect. FDO at 50 µM concentration remarkably increased the expression of different CYPs and, as a consequence, altered the cellular xenobiotic-metabolizing capacity. To the best of our knowledge, the impact of FDO on *CYP1A1* and *CYP1A2* expression and EROD activity has not been shown before, but it was shown by Wetmore et al. (2014) that FDO is mainly metabolized by CYP1A2. Effects of DIF, PPC and TBC on *CYP1A1* and/or *CYP1A2* expression or activity have been demonstrated previously (Egaas et al. 1999; Knebel et al. 2019b; Yang et al. 2018; Zhang et al. 2017). An increased metabolism of triazoles might be the underlying cause for enhanced steatotic effects, if not the parental triazole compounds, but their metabolites are the causative agents for steatosis. Up till now, however, it is not known whether the parental triazole compounds or their metabolites cause triglyceride accumulation in liver cells. The fact that NR activation by triazoles, the MIE of the steatosis AOP, was observed in HepG2 cells which are barely able to metabolize most xenobiotic compounds (including a lack of substantial expression of *CYP3A4*) (Luckert et al. 2017), argues for a substantial role of the non-metabolized compounds in causing steatosis. To elucidate if toxicokinetic interactions may be responsible for the potentiation effect, measurements of DIF in the supernatant of the culture medium were performed. It was observed that DIF disappeared at a slower rate from the supernatant in the presence of FDO, indicating decreased metabolism of the compound. This could be an indication for a toxicokinetic effect, as probably the metabolism or uptake of DIF is inhibited by FDO. It has been demonstrated that DIF, PPC and TBC are preferentially metabolized by CYP3A4 (Habenschus et al. 2019; Mazur and Kenneke 2008; Wetmore et al. 2014). For this reason, we investigated if FDO inhibits CYP3A4 enzyme activity. Indeed, FDO suppressed CYP3A4 enzymatic activity. This provides a mechanistic explanation for the observed diminished decrease of DIF levels when administered in mixtures, and in consequence the prolonged exposure of cells to higher concentrations of DIF can explain the phenomenon of increased triglyceride accumulation in HepaRG cells treated with a mixture of DIF and FDO.

In summary, a more than additive performance of in vitro liver triglyceride accumulation has been observed for the combination of steatosis-inducing triazole fungicides and non-steatotic compound FDO. However, future studies are necessary to confirm the relevance of the present in vitro findings in living organisms. Nonetheless, the present data clearly show that mixtures of compounds that do not share the same AO are capable of causing unexpected molecular effects. It appears that in the recent past, researchers have tended to focus on mixture effects related to toxicodynamic interactions, especially of compounds with shared AO and similar molecular mode(s) of action. Data from this study demonstrate that also toxicokinetic interactions have to be considered, and that the analysis of mixture effects should not be restricted to mixtures of compounds with obvious similarities of structures and toxic effects.

## Supplementary Information

Below is the link to the electronic supplementary material.

Supplementary file1 (DOCX 1725 KB)

## Abbreviations

AHR: Aryl hydrocarbon receptor
AO: Adverse outcome
AOP: Adverse outcome pathway
CA: Concentration addition concept
CAG: Cumulative assessment group
CAR: Constitutive androstane receptor
CI: Combination index
EC_50_: Half-maximal effective concentration
ER: Estrogen receptor
EROD: Ethoxyresorufin-*O*-deethylase
DIF: Difenoconazole
FDO: Fludioxonil
FXR: Farnesoid-X-receptor
GR: Glucocorticoid receptor
IA: Independent action concept
KE: Key event
KTC: Ketoconazole
LXR: Liver-X-receptor
MDR: Model deviation ratio
MIE: Molecular initiating event
NR: Nuclear receptor
PPAR: Peroxisome proliferator-activated receptor
PPC: Propiconazole
PXR: Pregnane-X-receptor
RAR: Retinoic acid receptor
RXR: Retinoid X receptor
TA: Theoretical additivity method
TBC: Tebuconazole
