# Supplementary material for: More than additive effects on liver triglyceride accumulation by combinations of steatotic and non-steatotic pesticides in HepaRG cells
Source: Arch Toxicol. 2021 Feb 11;95(4):1397–411. doi: 10.1007/s00204-021-02997-2 (PMC8032629; doi:10.1007/s00204-021-02997-2)
Supplement: Supplementary file 1 — Supplementary file1 (DOCX 1725 KB) [file 204_2021_2997_MOESM1_ESM.docx]

**More than additive effects on liver triglyceride accumulation by combinations of steatotic and non-steatotic pesticides in HepaRG cells**

*Alexandra Lasch^1^, Philip Marx-Stoelting^2^, Albert Braeuning^1*^, Dajana Lichtenstein^1^*

**Author information:**

^1^German Federal Institute for Risk Assessment, Department of Food Safety, Max-Dohrn-Straße 8-10, 10589 Berlin, Germany

^2^German Federal Institute for Risk Assessment, Department of Pesticides Safety, Max-Dohrn-Straße 8-10, 10589 Berlin, Germany

*Corresponding Author: Albert.Braeuning@bfr.bund.de; German Federal Institute for Risk Assessment, Department of Food Safety, Max-Dohrn-Straße 8-10, 10589 Berlin, Germany

**Supplementary Data**

**Fig. S1** Comparison of the normalization of the gene expression data to the mean of reference genes and to *ACTB* and *GAPDH*. As an example, one biological replicate of *CYP1A2* gene expression results is shown for single compounds DIF, PPC and TBC and their mixtures with 50 µM FDO. No relevant changes in the relative expression of *CYP1A2* occur, depending on the used reference gene.

**Fig. S2** Full concentration-response curves for triglyceride accumulation in HepaRG cells of mixtures of DIF (a, b, c), PPC (d, e, f) and TBC (g, h, i) with 50 µM FDO shown in black and compared to calculated mixture effect with TA (a, d, g), IA (b, e, h) and CA (c, f, i) shown in white. Data represent mean ± SD (N=3 independent experiments). Statistics was done by one-Way ANOVA with Holm-Sidak post-hoc test (all pairwise): * p ≤ 0.05, ** p ≤ 0.01 and *** p ≤ 0.001

**Fig. S3** EROD activity after 24 h incubation in HepaRG cells with medium control and 50 µM FDO. 3 µM 3-methylcholantrene was used as positive control (data not shown). Data represent means ± SD (N=3 independent experiments). Statistic was done by one-way ANOVA with Holm-Sidak post-hoc test (all pairwise): *** p ≤ 0.001

**Fig. S4** Gene expression results after 24 h incubation in HepaRG cells by mixtures of DIF, PPC and TBC with 50 µM FDO and by single substances. Illustrated are the genes with the most striking mixtures effect: (a) *CYP1A1*, (b) *CYP1A2*, (c) *CYP2B6*, (d) *CAR* and (e) *ACOX2*. Data represent mean ± SD (N=3 independent experiments). Statistic was done by one-way ANOVA with Dunnett‘s test, (* p ≤ 0.05, ** p ≤ 0.01 and *** p ≤ 0.001) against medium control

**Fig. S5** Western blots of ACOX2 knockdown via siRNA and incubation of HepaRG cells with 50 µM FDO. M= medium, NC = siRNA negative control transfection, A= ACOX2 siRNA, FDO = 50 µM fludioxonil

**Table S1** Mean and SD of NR activation results of FDO, DIF, PPC and TBC

|  |  | PXR | | LXR | | FXR | | RXR | | RAR | | AHR | | PPARa | | PPARg | | PPARd | | GR | | CAR | |
| --- | --- | --- | --- | --- | --- | --- | --- | --- | --- | --- | --- | --- | --- | --- | --- | --- | --- | --- | --- | --- | --- | --- | --- |
| substance | conc. µM | mean | SD | mean | SD | mean | SD | mean | SD | mean | SD | mean | SD | mean | SD | mean | SD | mean | SD | mean | SD | mean | SD |
| FDO | 1.00 | 1.18 | 0.17 | 1.24 | 0.34 | 1.78 | 0.98 | 1.35 | 0.18 | 1.11 | 0.39 | 1.42 | 0.17 | 0.87 | 0.12 | 1.03 | 0.19 | 1.27 | 0.40 | 1.02 | 0.49 | 1.31 | 0.06 |
|  | 25.00 | 1.66 | 0.47 | 0.26 | 0.16 | 0.92 | 0.72 | 8.37 | 1.74 | 0.35 | 0.15 | 3.86 | 1.32 | 0.69 | 0.21 | 1.57 | 0.54 | 1.42 | 0.64 | 0.48 | 0.09 | 0.31 | 0.10 |
|  | 50.00 | 7.96 | 3.73 | 0.21 | 0.23 | 0.66 | 0.62 | 7.98 | 3.35 | 0.15 | 0.10 | 3.67 | 0.96 | 0.66 | 0.18 | 0.85 | 0.13 | 0.86 | 0.09 | 0.39 | 0.19 | 0.29 | 0.07 |
|  | 100.00 | 9.70 | 5.37 | 0.11 | 0.08 | 0.31 | 0.11 | 7.20 | 1.92 | 0.10 | 0.03 | 3.32 | 1.03 | 0.61 | 0.21 | 0.61 | 0.05 | 0.81 | 0.12 | 0.38 | 0.13 | 0.17 | 0.05 |
| DIF | 0.10 | 1.53 | 0.59 | 2.44 | 1.34 | 0.88 | 0.07 | 1.11 | 0.26 | 0.67 | 0.13 | 0.92 | 0.14 | 0.75 | 0.05 | 0.95 | 0.10 | 1.01 | 0.12 | 0.93 | 0.09 | 0.78 | 0.06 |
|  | 1.00 | 2.62 | 1.58 | 3.79 | 3.02 | 0.83 | 0.15 | 0.87 | 0.25 | 0.55 | 0.15 | 1.19 | 0.11 | 0.88 | 0.20 | 1.06 | 0.04 | 0.97 | 0.12 | 0.92 | 0.13 | 0.50 | 0.05 |
|  | 3.00 | 3.01 | 1.83 | 3.06 | 2.51 | 0.94 | 0.03 | 0.71 | 0.13 | 0.46 | 0.15 | 1.27 | 0.09 | 0.73 | 0.10 | 1.10 | 0.20 | 0.99 | 0.16 | 0.90 | 0.17 | 0.27 | 0.03 |
|  | 5.00 | 2.80 | 1.70 | 2.90 | 2.19 | 1.35 | 0.23 | 0.53 | 0.09 | 0.37 | 0.06 | 1.37 | 0.15 | 0.50 | 0.10 | 0.99 | 0.16 | 0.76 | 0.04 | 0.99 | 0.20 | 0.17 | 0.04 |
| PPC | 5.00 | 1.13 | 0.17 | 1.01 | 0.25 | 0.77 | 0.18 | 0.96 | 0.20 | 3.47 | 0.15 | 2.38 | 0.44 | 0.91 | 0.24 | 0.81 | 0.03 | 0.71 | 0.21 | 0.90 | 0.17 | 2.56 | 0.46 |
|  | 10.00 | 2.27 | 0.48 | 1.07 | 0.19 | 0.93 | 0.29 | 1.03 | 0.36 | 5.28 | 1.31 | 3.63 | 0.95 | 0.84 | 0.24 | 1.03 | 0.05 | 0.96 | 0.21 | 0.91 | 0.11 | 3.44 | 0.25 |
|  | 20.00 | 5.55 | 0.78 | 0.72 | 0.22 | 0.78 | 0.22 | 0.92 | 0.28 | 7.40 | 1.26 | 6.25 | 2.24 | 0.79 | 0.29 | 1.03 | 0.10 | 0.82 | 0.18 | 1.09 | 0.38 | 4.82 | 1.16 |
|  | 40.00 | 14.98 | 3.74 | 0.70 | 0.19 | 1.13 | 0.52 | 0.59 | 0.24 | 10.00 | 3.44 | 12.34 | 4.11 | 0.57 | 0.32 | 1.30 | 0.27 | 0.94 | 0.10 | 1.59 | 0.68 | 5.18 | 1.82 |
| TBC | 5.00 | 1.39 | 0.15 | 0.90 | 0.11 | 0.82 | 0.07 | 0.77 | 0.07 | 1.22 | 0.22 | 1.23 | 0.28 | 0.69 | 0.16 | 0.96 | 0.09 | 1.00 | 0.09 | 1.20 | 0.45 | 0.69 | 0.17 |
|  | 10.00 | 2.42 | 1.14 | 0.94 | 0.29 | 0.89 | 0.10 | 0.70 | 0.12 | 1.18 | 0.11 | 1.53 | 0.42 | 0.74 | 0.07 | 0.85 | 0.10 | 1.00 | 0.08 | 0.88 | 0.04 | 0.59 | 0.05 |
|  | 20.00 | 4.46 | 1.81 | 1.18 | 0.17 | 0.79 | 0.06 | 0.56 | 0.06 | 1.42 | 0.23 | 1.44 | 0.28 | 0.56 | 0.13 | 0.74 | 0.14 | 0.84 | 0.32 | 0.77 | 0.09 | 0.45 | 0.05 |
|  | 40.00 | 6.20 | 1.93 | 1.92 | 1.03 | 0.76 | 0.11 | 0.54 | 0.12 | 1.42 | 0.16 | 1.75 | 0.43 | 0.49 | 0.14 | 0.78 | 0.12 | 0.91 | 0.22 | 0.60 | 0.25 | 0.28 | 0.03 |

**Table S2:** Western blot data for 50 µM FDO and ACOX2 knockdown via siRNA

| **treatment** | **incubation time** | **ACOX2 Volume (Int)** | **PAN-Actin Volume (Int)** | **ACOX2/Actin** | **biol. replicate** |
| --- | --- | --- | --- | --- | --- |
| medium | 24 h | 7741763 | 13785759 | 0.56157684 | 1 |
| si-NC TF | 24 h | 11350409 | 19057528 | 0.59558664 |  |
| si-ACOX2 TF | 24 h | 7851760 | 21351762 | 0.36773359 |  |
|  |  |  |  |  |  |
| medium | 96 h | 12000066 | 16087734 | 0.745914 |  |
| si-NC TF | 96 h | 10571444 | 13581026 | 0.77839804 |  |
| si-ACOX2 TF | 96 h | 5836992 | 13062322 | 0.44685715 |  |
|  |  |  |  |  |  |
| medium | 48 h | 12963376 | 19293474 | 0.67190471 |  |
| FDO 50 µM | 24 h | 14808864 | 14699559 | 1.00743594 |  |
| FDO 50 µM | 48 h | 7963650 | 18762848 | 0.42443716 |  |
|  |  |  |  |  |  |
| **treatment** | **incubation time** | **ACOX2 Volume (Int)** | **PAN-Actin Volume (Int)** | **ACOX2/Actin** | **biol. replicate** |
| medium | 24 h | 4048617 | 8537564 | 0.4742122 | 2 |
| si-NC TF | 24 h | 4989216 | 11693232 | 0.42667553 |  |
| si-ACOX2 TF | 24 h | 4715919 | 13378167 | 0.35250861 |  |
|  |  |  |  |  |  |
| medium | 96 h | 6563942 | 11539617 | 0.56881801 |  |
| si-NC TF | 96 h | 6974100 | 12555912 | 0.55544352 |  |
| si-ACOX2 TF | 96 h | 4790688 | 14055600 | 0.34083838 |  |
|  |  |  |  |  |  |
| medium | 48 h | 14527900 | 20280715 | 0.71634062 |  |
| FDO 50 µM | 24 h | 14432936 | 16426575 | 0.87863331 |  |
| FDO 50 µM | 48 h | 5742950 | 11437362 | 0.50212191 |  |
|  |  |  |  |  |  |
|  |  |  |  |  |  |
| **treatment** | **incubation time** | **ACOX2 Volume (Int)** | **PAN-Actin Volume (Int)** | **ACOX2/Actin** | **biol. replicate** |
| medium | 24 h | 9867250 | 14250720 | 0.69240361 | 3 |
| si-NC TF | 24 h | 14596524 | 14160805 | 1.03076937 |  |
| si-ACOX2 TF | 24 h | 14198859 | 15615761 | 0.90926462 |  |
|  |  |  |  |  |  |
| medium | 96 h | 14374605 | 16236564 | 0.88532309 |  |
| si-NC TF | 96 h | 17839906 | 19103418 | 0.93385938 |  |
| si-ACOX2 TF | 96 h | 7204600 | 18726741 | 0.38472257 |  |
|  |  |  |  |  |  |
| medium | 48 h | 14846312 | 10899148 | 1.36215345 |  |
| FDO 50 µM | 24 h | 14555691 | 13496980 | 1.07844058 |  |
| FDO 50 µM | 48 h | 11126336 | 15189100 | 0.73252108 |  |
